# P-2274. Incidence and Microbiology of Postoperative Pleural Space Infections among Lung Transplant Recipients

**DOI:** 10.1093/ofid/ofae631.2427

**Published:** 2025-01-29

**Authors:** Kellie J Goodlet, Michael D Nailor, Sofya Tokman

**Affiliations:** Midwestern University College of Pharmacy; St. Joseph’s Hosp. and Med. Ctr., Phoenix, AZ; Norton Thoracic Institute, St. Joseph’s Hospital and Medical Center, Phoenix, Arizona

## Abstract

**Background:**

Pleural space infections (PSI) represent a known complication of lung transplantation (LT) in the early posttransplant period. Within the historical literature, PSIs, and empyemas in particular, have been associated with an increased risk for death, with 29-43% 1-year mortality. However, these studies were small and conducted prior to advances in anti-infective prophylaxis and treatment strategies. Therefore, the purpose of this investigation was to characterize the microbial profile and outcomes of early PSI among lung transplant recipients (LTRs) within the modern era.

**Figure 1.**
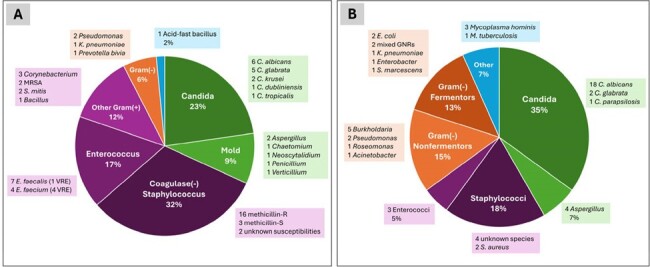
Isolated organisms from pleural fluid of lung transplant recipients from present study (Panel A, 66 isolates, 2017-2022 transplants) and historical literature (Panel B, 4 studies, 55 isolates, 1983-2005 transplants)

**Methods:**

This study was approved by the Institutional Review Board of St. Joseph’s Hospital and Medical Center (SJHMC). Included LTRs were transplanted between 2017-2022 at SJHMC with ≥ 1 pleural fluid culture positive for an organism within 90 days of LT. LTRs at SJHMC received cefepime, vancomycin, and levofloxacin in the immediate postoperative period, or targeted antibiotic therapy based on donor and recipient airway cultures. During the index transplant hospitalization, LTRs received prophylaxis with inhaled amphotericin and tobramycin. Additional opportunistic infection prophylaxis throughout the study period included valganciclovir, itraconazole, and trimethoprim/sulfamethoxazole.

**Figure:**
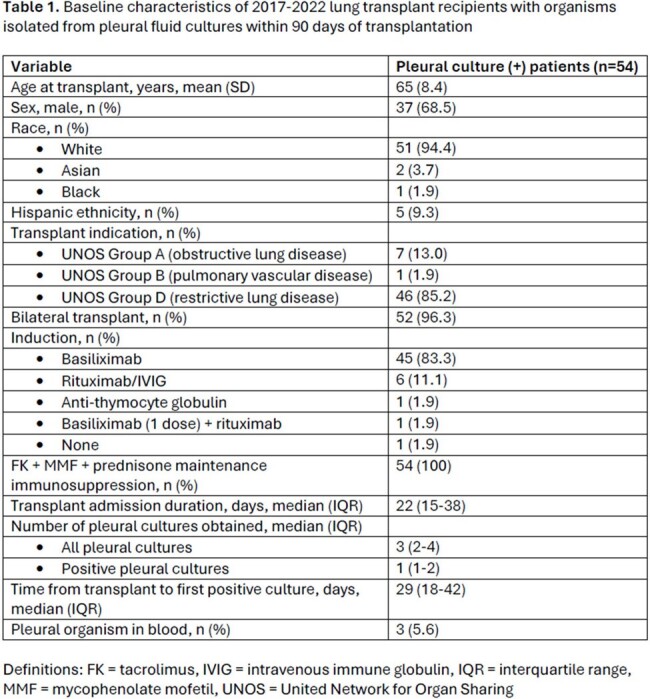

**Results:**

Among 554 total LTRs, 250 (45%) had pleural effusion cultures, and 54 (54/250, 22%) had ≥ 1 pleural culture positive for an organism (Table 1). Over 95% of effusions were exudative and 53% were neutrophil predominant. Of the 66 organisms isolated (Figure 1), 40 (61%) were Gram-positive bacteria, 21 (32%) were fungi (15 *Candida* species, 6 molds), 4 (6%) were Gram-negative bacteria, and 1 (2%) was unspeciated acid-fast bacilli. Infection was typically managed via effusion drainage and 4-6 weeks of pathogen-directed antimicrobial therapy. All-cause 1-year mortality was 7.4%, lower than national LTR 1-year mortality rates over the study period (∼10-12%).

**Conclusion:**

In a large investigation of LTRs with early posttransplant PSIs, Gram-positive bacteria were isolated most frequently, with isolation of *Candida* also common. Gram-negative bacteria comprised a smaller proportion of infections than reported in prior literature. PSIs did not appear to predispose LTRs to poor posttransplant survival.

**Disclosures:**

Michael D. Nailor, Pharm.D., InflaRx: Advisor/Consultant|Shionogi: Advisor/Consultant

